# pEPito: a significantly improved non-viral episomal expression vector for mammalian cells

**DOI:** 10.1186/1472-6750-10-20

**Published:** 2010-03-15

**Authors:** Rudolf Haase, Orestis Argyros, Suet-Ping Wong, Richard P Harbottle, Hans J Lipps, Manfred Ogris, Terese Magnusson, Maria G Vizoso Pinto, Jürgen Haas, Armin Baiker

**Affiliations:** 1 Max von Pettenkofer-Institute, University of Munich, Munich, Germany; 2 National Heart and Lung Institute, Imperial College London, London, UK; 3 Institute for Cell Biology, University of Witten/Herdecke, Witten, Germany; 4 Department of Pharmacy, University of Munich, Munich, Germany; 5 Division of Pathway Medicine, University of Edinburgh, Edinburgh, UK

## Abstract

**Background:**

The episomal replication of the prototype vector pEPI-1 depends on a transcription unit starting from the constitutively expressed *Cytomegalovirus *immediate early promoter (CMV-IEP) and directed into a 2000 bp long *matrix attachment region sequence *(MARS) derived from the human β-interferon gene. The original pEPI-1 vector contains two mammalian transcription units and a total of 305 CpG islands, which are located predominantly within the vector elements necessary for bacterial propagation and known to be counterproductive for persistent long-term transgene expression.

**Results:**

Here, we report the development of a novel vector pEPito, which is derived from the pEPI-1 plasmid replicon but has considerably improved efficacy both *in vitro *and *in vivo*. The pEPito vector is significantly reduced in size, contains only one transcription unit and 60% less CpG motives in comparison to pEPI-1. It exhibits major advantages compared to the original pEPI-1 plasmid, including higher transgene expression levels and increased colony-forming efficiencies *in vitro*, as well as more persistent transgene expression profiles *in vivo*. The performance of pEPito-based vectors was further improved by replacing the CMV-IEP with the *human CMV enhancer/human elongation factor 1 alpha promoter *(hCMV/EF1P) element that is known to be less affected by epigenetic silencing events.

**Conclusions:**

The novel vector pEPito can be considered suitable as an improved vector for biotechnological applications *in vitro *and for non-viral gene delivery *in vivo*.

## Background

**Figure 1:**
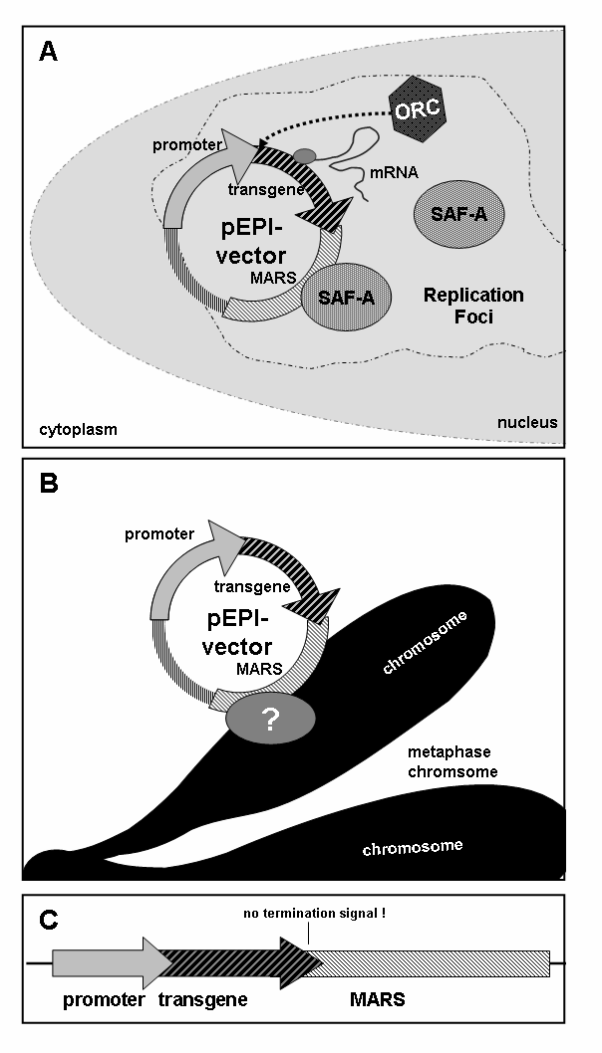
**Mechanism of persistence of MARS-containing pEPI-1 vectors**. (**A**) Episomal replication can be explained by a stable association with early replication foci mediated by the MARS mediated binding to the nuclear matrix protein *scaffold attachment factor A *(SAF-A). Within early replication foci, the assembly of the origin recognition complex (ORC) and the DNA replication of pEPI vector molecules is likely facilitated by a conformational change resulting from mRNA transcription.(**B**) Nuclear retention and mitotic stability of pEPI-based vectors can be explained by MARS mediated direct or indirect interaction with metaphase chromosomes in a "piggy back"-like mechanism. (**C**) The functional element of pEPI-vector plasmid replicons consists of a transcription unit, regulated by a constitutive promoter and directed into a chromosomal MARS with the prerequisite of no termination signal being located between transcription unit and MARS element.

The non-viral plasmid vector pEPI-1 was constructed in 1999 by Piechaczek et al [1], by cloning a 2000 bp long *matrix attachment region sequence *(MARS), that was derived of the human β-interferon gene cluster [2], into the commercial vector pGFP-C1 (Clontech, USA). The pEPI-1 vector contains a total of 305 CpG motives, most of them within the vector elements necessary for bacterial propagation. Furthermore, pEPI-1 consists of two mammalian transcription units oriented in a clockwise direction. The first, *Cytomegalovirus immediate early promoter *(CMV-IEP) driven *enhanced green fluorescent protein *(EGFP) transcription unit is oriented into the MARS and has been shown to be a functional component of the pEPI-1 vector plasmid replicon. The second, *Simian Virus 40 Ori/promoter *(SV40-O/P) driven *neomycin phosphotransferase *(NPT) transcription unit has been used for bacterial and mammalian selection purposes, but is dispensable for the vectors' episomal replication in mammalian cells. pEPI-1 replicates episomally in a copy number of approximately 5-10 molecules per cell in all mammalian cell lines tested, is mitotically stable even in the absence of selection and facilitates long-term expression of transgenes or shRNA's [1,3-5]. The vector replicates once per cell cycle during early S-phase, with the *origin recognition complex *(ORC) being able to assemble at various regions on the vector DNA [6]. The episomal replication is due to stable association with early replication foci by MARS mediated binding to the nuclear matrix protein *scaffold attachment factor A *(SAF-A). Within early replication foci, DNA replication of pEPI-1 vector molecules is likely facilitated by a conformational change resulting from mRNA transcription [3,7,8] (Figure 1A). The mitotic stability of pEPI-based vectors can be explained by the MARS mediated stable interaction with metaphase chromosomes [3] (Figure 1B). The functional element of the pEPI-1 plasmid replicon could be mapped as transcription unit, regulated by a constitutive promoter and directed into a chromosomal MARS without termination signal between transcription unit and MARS [9] (Figure 1C).

Similar to EBV plasmid replicons administered as naked DNA, the establishment of stable pEPI-1 episomes in transfected cells is very inefficient [1,10]: only 0.5-5% of transiently pEPI-1-transfected cells develop stable clones. This is also the case when pEPI-1 plasmids are isolated from already established mammalian cell clones and reintroduced into cells demonstrating that the primary sequence of DNA is not involved in the establishment process [8,11,12] but rather epigenetic features such as chromatin structure and nuclear localization [8].

A *cytosine monophosphate *(C) followed by a *guanine monophosphate *(G) in a nucleotide sequence is referred to as CpG dinucleotide (or CpG islands). In eukaryotic DNA, CpG dinucleotides in eukaryotes appear at lower frequency and are often methylated to *5-methyl-cytosine *(^m^CpG), whereas in most bacterial genomes, however, CpG dinucleotides are represented according to their statistical expectation, and the cytosine residue is normally unmethylated [13,14]. The human innate immune system has evolved mechanisms to differentiate bacterial DNA from its own via *Toll-like receptor 9 *(TLR 9) signalling. TLR9 interacts with endocytosed DNA comprising unmethylated CpG dinucleotides and triggers downstream signalling via MyD88, IRAK and TRAF6 to increase NFêB and AP1 expression. This in turn results in the production of inflammatory cytokines [15,16]. In fact, Hyde et al. demonstrated that a single CpG motif present in a DNA vector backbone elicits an inflammation after pulmonary delivery *in vivo *[17].

The majority of human promoters belong to the high-CpG class of promoters, for which it is well established that the methylation status of the surrounding CpG islands has a direct influence on the promoter activity unmethylated CpG islands in the promoter region correspond to active promoters, whereas heavily methylated ^m^CpG islands correspond to inactive promoters [18]. The CMV-IEP is most often used in commercial vectors conferring robust expression of a transgene in several cell types. This strong activity is due to the presence of several transcription factor binding sites within its sequence. The expression profile of CMV-IEP typically peaks at around 1-2 days after vector administration, followed by a steady decrease in its activity over a period of 1-2 weeks. Methylation of CpG islands within the CMV-IEP region has been suggested as one explanation for this long-known, but undesired silencing phenomenon [19], even if recent publications indicate other mechanisms for this effect [20,21].

Taken together, these findings suggest that a state of the art plasmid vector for gene delivery into mammalian cells should be composed of a promoter element that is depleted of CpG islands in order to minimize silencing phenomena, and a CpG depleted vector backbone to prevent undesired stimulatory effects on the innate immune system [16,22]. Such optimized plasmid vectors have already been realized as *CpG depleted vectors *[17,23] exhibiting increased levels and persistence of transgene expression, as well as reduced inflammation *in vivo*.

In the present study we hypothesized that pEPI-1 based plasmid replicons can be improved by reducing the CpG content in the vector backbone to achieve more efficient transgene expression *in vitro *and *in vivo*. The novel vector named pEPito has been constructed by cloning the pEPI-1 plasmid replicon into a plasmid backbone without CpG islands and omitting a second transcription unit.

Herein we demonstrate that the novel pEPito vector exhibits several advantages over its precursor pEPI-1, including higher transgene expression levels and colony-forming efficiencies *in vitro *and more persistent transgene expression profiles *in vivo*. Additionally, the performance of all vectors *is *improved when replacing the CMV-IEP by the *human CMV enhancer/human elongation factor 1 alpha promoter *(hCMV/EF1P) element, that is known to be less affected by epigenetic silencing events [17].

## Results

### Generation of pEPI-based vectors with improved efficacy

**Figure 2:**
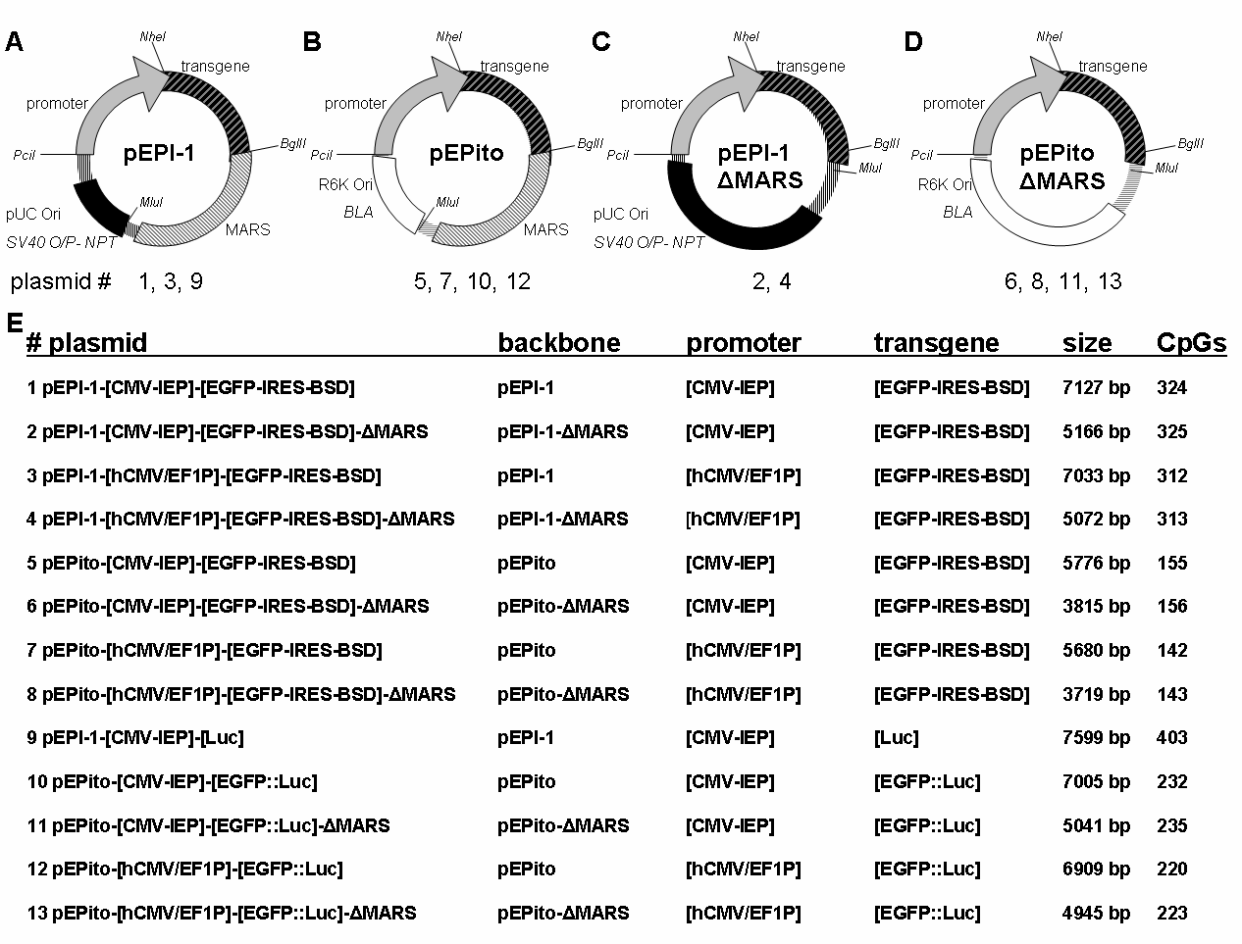
**pEPI-derived plasmids generated in this study**. A schematic picture of the four types of vector backbones is depicted in the upper panel: pEPI-1 (**A**), pEPito (**B**), pEPI-1-ΔMARS (**C**), or pEPito-ΔMARS (**D**). All vectors contain either the CMV-IEP or the hCMV/EF1P promoter element. Depending on the experiment, three different transgenes have been used: an *EGFP-BSD cassette connected via an internal ribosomal entry site module *(EGFP-IRES-BSD), a *Firefly luciferase *(Luc), or an *EGFP-luciferase fusion protein *(EGFP::Luc). All 13 different vectors have been constructed, propagated, and amplified in *E.coli *DB3.1λpir. (**E**) Additional information regarding the different vectors. The vector pEPI-1- [CMV-IEP]- [Luc] has been recently published as pEPI-Luc [32]. This figure does not claim proportional correctness.

We systematically generated a panel of 13 different pEPI-1-derived vectors, all of them constructed in a modular way enabling the easy exchange of backbone, promoter, or transgene elements by restriction digest with *Pci*I, *Nhe*I, *Bgl*II, or *Mlu*I (Figure 2). Vectors based on the pEPI-1 backbones contain a pUC Ori for bacterial propagation, a MARS, a second mammalian SV40-O/P driven NPT transcription unit for bacterial kanamycin or mammalian *geneticin *(G418) selection purposes, and a total of 206 CpG motives. The pEPito backbones contain a R6K Ori [24] for bacterial propagation, a *β-lactamase gene *(BLA) for bacterial ampicillin selection, a MARS, no second mammalian transcription unit, and only 37 CpG motives. Control vectors pEPI-1-ΔMARS and pEPito-ΔMARS without MARS elements contain a total of 207 and 38 CpG motives, respectively. All vectors contain either the CMV-IEP, or the hCMV/EF1P promoter element, with the latter one known to be less affected by epigenetic silencing events [17]. Depending on the experiment, three different transgenes have been used: an *EGFP-BSD cassette with which both eGFP (enhanced green fluorescent protein) and BSD (Blasticidine S deaminase) are expressed via a synthetic internal ribosomal entry site *(EGFP-IRES-BSD) [25], a *Firefly luciferase *(Luc) [26,27], or an *EGFP-luciferase fusion protein *(EGFP::Luc) [27-29]. All 13 different vectors have been constructed, propagated, and amplified in *E.coli *DB3.1ëpir [30].

### Increased transgene expression and colony formation of pEPito vectors *in vitro*

In order to compare the novel CpG depleted pEPito vector backbone with the original pEPI-1 vector backbone, a set of eight novel vectors were constructed (Figure 2E, #1-8). All novel vectors comprised an identical transgene, i.e. an EGFP-BSD cassette connected via a recently described, 22 bp long internal ribosomal entry site (EGFP-IRES-BSD) [25]. This cassette enabled both the analysis of transgene (EGFP) expression by flow cytometry and the selection of transfected mammalian cells with blasticidin.

**Figure 3:**
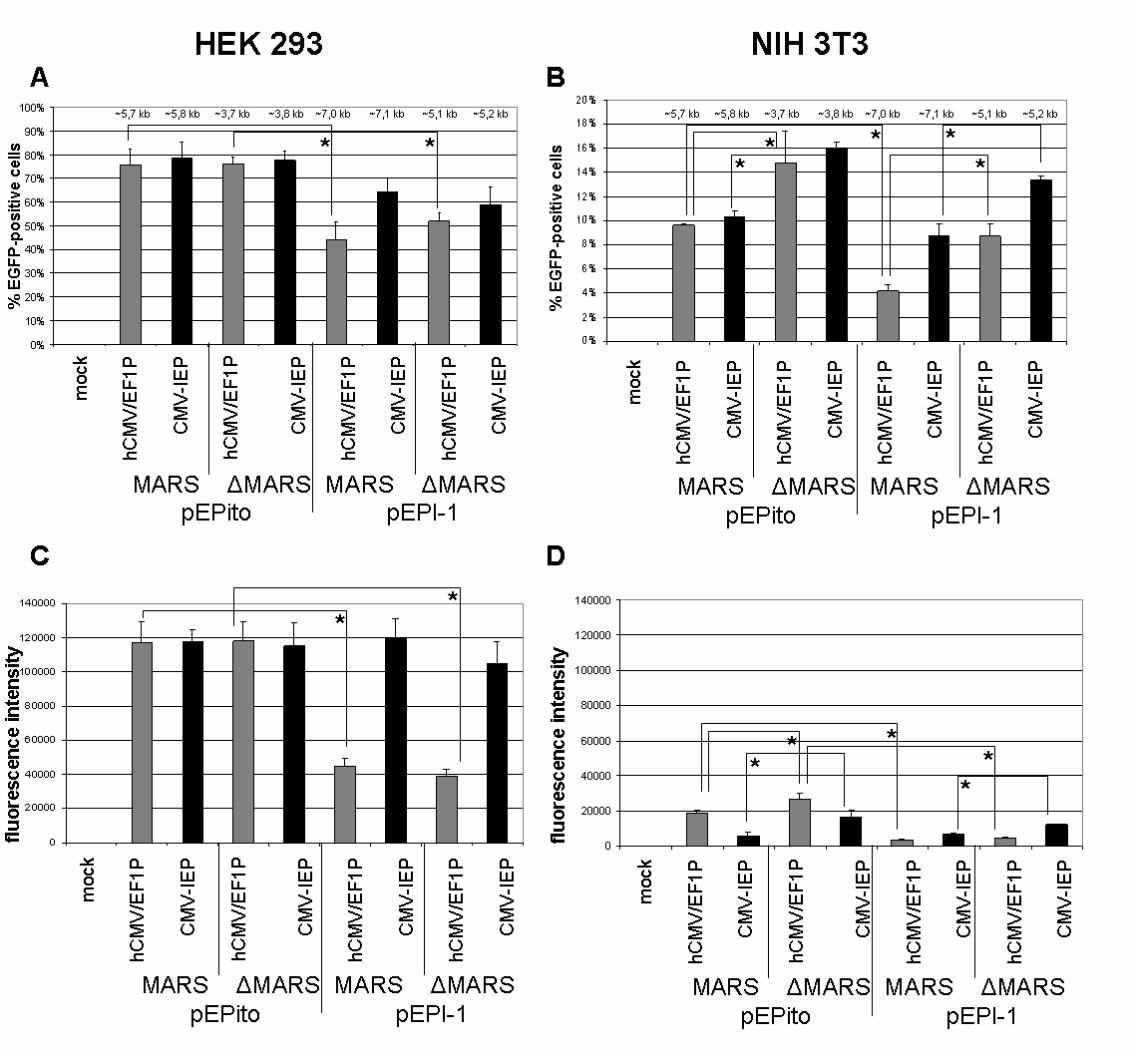
**Transfection efficiencies and expression levels in transiently transfected HEK293 and NIH3T3 cells**. Transient transfection efficiencies (**A, B**) and the transient mean EGFP expression levels per cell (**C, D**) obtained by transfection of the vectors #1-8 into HEK293 cells (**A, C**), or NIH3T3 cells (**B, D**). All vectors contain the EGFP-IRES-BSD transgene transcription unit. Mean values of n = 8 derived from four independent experiments ± SD are shown; * p < 0.05; ** p < 0.001 (two-tailed Student's *t*-test). mock: untransfected control. Grey bars indicate vectors containing an hCMV/EF1P promoter element, black bars indicate vectors containing a CMV-IEP promoter element.

I*n vitro *experiments were performed in HEK293 and NIH3T3 cells, with the latter cell line having shown to silence exogenous DNA rapidly [31] (Figure 3). Equal amounts of vector DNA were used to exclude undesired effects of any added stuffer DNA, as for example sonicated salmon sperm DNA or any other small plasmid DNA with varying CpG contents. Transient transfection results assayed by flow cytometry of EGFP positive cells 48 hours post transfection are shown (for representative FACS diagrams see additional file 1). On HEK293 cells, all pEPito-based vectors exhibited transfection efficiencies of around 75%, independent of plasmid size, promoter type, or the presence or absence of the MARS element, whereas with all pEPI-1 based vectors 45-65% EGFP positive cells were found (Figure 3A). pEPI-1 based vectors with hCMV/EF1P promoter element resulted in the lowest transient transfection efficiencies, indicating an effect of the used promoter elements. When performing identical experiments on NIH3T3 cells, overall lower transient transfection efficiencies were observed, ranging from 4% to 16% (Figure 3B). The pEPito-ΔMARS vectors, the smallest vectors used, exhibited the highest transient transfection efficiency indicating either an inverted effect of the plasmid size, or an effect of the initial molar amount of DNA used for transfection or both. In pEPI-1 based vector constructs the hCMV/EF1P promoter element exhibit lower transient transfection efficiencies compared to the CMV-IEP promoter element, and smaller, MARS depleted vectors exhibit higher transfection efficiencies compared to the larger plasmid constructs.

When analyzing the level of EGFP per cell, the mean EGFP intensity levels HEK293 cells were not significantly different in six out of the eight novel vector constructs (Figure 3C). Only pEPI-1- [hCMV/EF1P]- [EGFP-IRES-BSD] and pEPI-1- [hCMV/EF1P]- [EGFP-IRES-BSD]-ΔMARS resulted in a lower EGFP signal indicating an effect of the promoter elements only within the pEPI-1 vector backbone (Figure 3C). In NIH3T3 cells, pEPito based vectors with hCMV/EF1P promoter resulted in slightly higher EGFP expression levels as compared to the CMV-IEP (Figure 3D). In contrast, pEPI-based vectors with CMV-IEP promoter always resulted in slightly higher expression levels as the respective hCMV/EF1P promoter constructs. In concordance with transfection efficiencies, the overall mean intensity of the EGFP signal, however, was much weaker in NIH3T3 cells than in HEK293.

**Figure 4:**
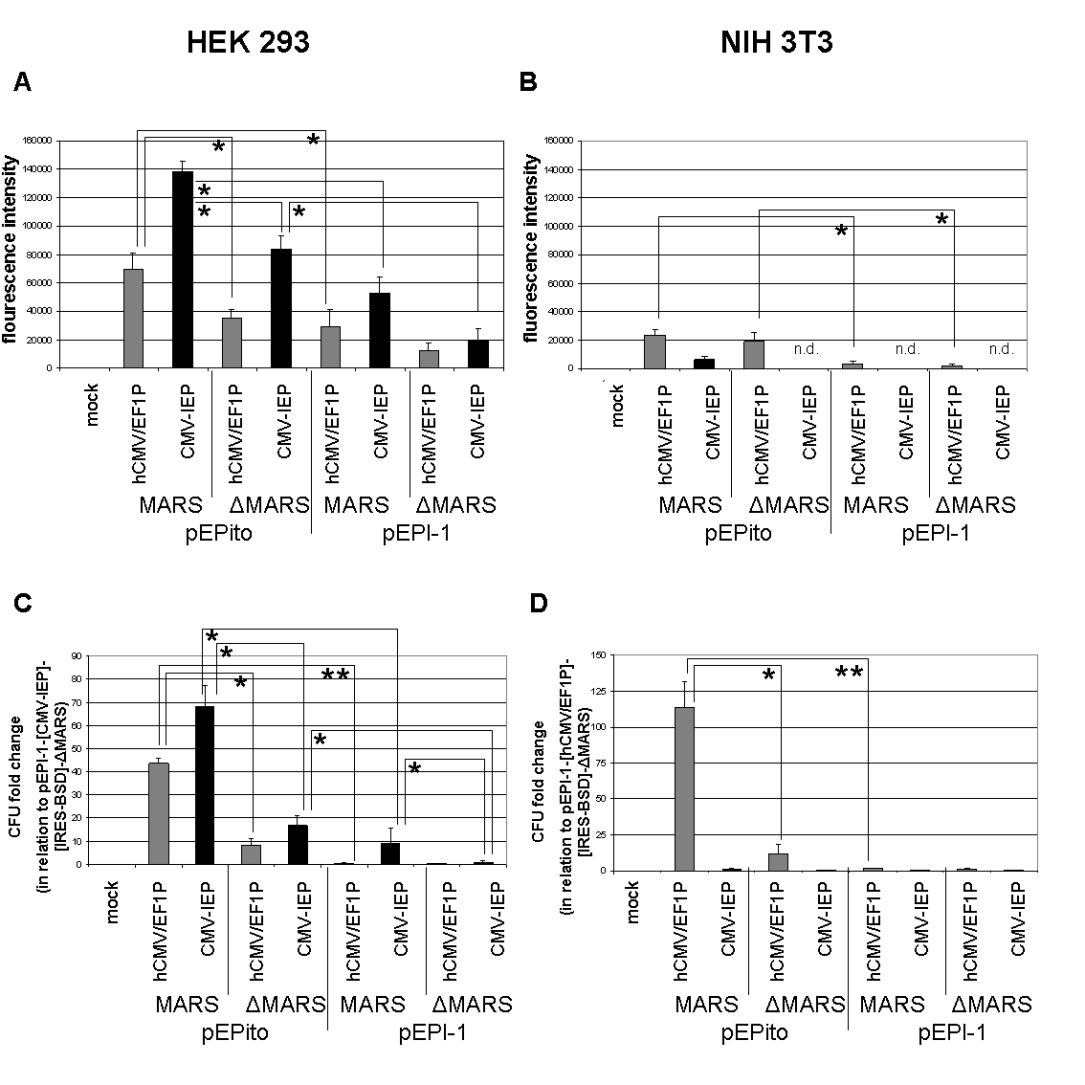
**Expression levels and colony formation efficiencies in stably selected HEK293 and NIH3T3 cells**. (**A, B**) Mean EGFP expression levels per cell (A: HEK293, B: NIH3T3) of stably selected cells. (**C, D**) Relative colony-forming efficiency of stably selected HEK293 cells (**C, **normalized to pEPI-1- [CMV-IEP]- [EGFP-IRES-BSD]-ΔMARS) or NIH3T3 cells (**B, **normalized to pEPI-1- [hCMV/EF1P]- [EGFP-IRES-BSD]-ΔMARS. All vectors contain the EGFP-IRES-BSD transgene transcription unit. Mean values of n = 8 derived from four independent experiments ± SD are shown; * p < 0.05; ** p < 0.001 (two-tailed Student's *t*-test). mock: untransfected control. n.d.: no stable mixed-clones obtained after transfection and blasticidin selection. Grey bars: vectors containing the hCMV/EF1 promoter element; black bars: vectors containing the CMV-IEP promoter element.

For further analysis, stably selected mixed-clone cells were generated from initially transfected cells as batch by selection with blasticidin for 30 days. When selecting HEK293 cells transfected with the eight different vector constructs, stably selected mixed-clone cells could be obtained in all cases (Figure 4A, C). In HEK293 cells the mean EGFP signal intensity per cell was influenced by the promoter element used, the nature of the vector backbone, and the presence or absence of a MARS. Vectors with the CMV-IEP promoter element exhibited higher EGFP expression levels as compared to their corresponding vectors with the hCMV/EF1P promoter element. Furthermore, pEPito-based vectors always exhibited higher expression levels compared to their respective pEPI-1-based counterparts. Finally, vectors with a MARS element always performed better than their MARS depleted controls, with respect to the EGFP expression level (Figure 4A, for representative FACS diagrams see additional file 2). With NIH3T3 cells, stably selected mixed-clone cells could not be obtained with the three CMV-IEP promoter element containing constructs indicating a negative effect of the CMV-IEP promoter element on the establishment of stable NIH3T3 cell lines (Figure 4B, D). The mean EGFP signal intensity per cell was mainly influenced by the promoter element and the nature of the vector backbone used, as the CpG depleted pEPito vectors always performed better than their pEPI-1-based counterparts. As most vectors based on the CMV-IEP promoter element could not be selected to obtain stable mixed-clones and that the vector pEPito- [CMV-IEP]- [EGFP-IRES-BSD] exhibits a weaker mean EGFP signal intensity then the vector pEPito- [hCMV/EF1P]- [EGFP-IRES-BSD], we conclude that the promoter element has a strong influence on the EGFP expression levels in NIH3T3 cells. In contrast to HEK293 cells, the promoter preference is inverted in NIH3T3 cells with the hCMV/EF1P always performing better than the CMV-IEP promoter element (Figure 4B).

The episomal status of all four MARS bearing vectors could be verified by bacterial rescue experiments using chromosomal DNA extracted from stably selected HEK293 mixed-clones. Rescued plasmids did not exhibit any rearrangements as checked by restriction analysis and gel electrophoresis (data not shown). The plasmids pEPito- [hCMV/EF1P]- [EGFP-IRES-BSD] and pEPito- [CMV-IEP]- [EGFP-IRES-BSD] could be rescued from chromosomal DNA extracted from stably selected NIH3T3 cells, but no MARS depleted control vector could be rescued (Table 1).

**Table 1:** Rescue of plasmids from stably transfected cells and injected mice

#	plasmid	HEK293 **30 d.p.t**.	NIH3T3 **30 d.p.t**.	mouse liver **32 d.p.i**.
1	pEPI-1- [CMV-IEP]- [EGFP-IRES-BSD]	**3/3**	**0/9**	n.a.

2	pEPI-1- [CMV-IEP]- [EGFP-IRES-BSD]-ΔMARS	**0/9**	**0/9**	n.a.

3	pEPI-1- [hCMV/EF1P]- [EGFP-IRES-BSD]	**3/3**	**-/9**	n.a.

4	pEPI-1- [hCMV/EF1P]- [EGFP-IRES-BSD]-ΔMARS	**-/9**	**-/9**	n.a.

5	pEPito- [CMV-IEP]- [EGFP-IRES-BSD]	**3/3**	**3/3**	n.a.

6	pEPito- [CMV-IEP]- [EGFP-IRES-BSD]-ΔMARS	**-/9**	**0/9**	n.a.

7	pEPito- [hCMV/EF1P]- [EGFP-IRES-BSD]	**3/3**	**3/3**	n.a.

8	pEPito- [hCMV/EF1P]- [EGFP-IRES-BSD]-ΔMARS	**-/9**	**-/9**	n.a.

9	pEPI-1- [CMV-IEP]- [Luc]	n.a.	n.a.	**-/9**

10	pEPito- [CMV-IEP]- [EGFP::Luc]	n.a.	n.a.	**3/3**

11	pEPito- [CMV-IEP]- [EGFP::Luc]-ΔMARS	n.a.	n.a.	**-/9**

12	pEPito- [hCMV/EF1P]- [EGFP::Luc]	n.a.	n.a.	**3/3**

13	pEPito- [hCMV/EF1P]- [EGFP::Luc]-ΔMARS	n.a.	n.a.	**-/9**

First column: consecutive vector numbers and systematic vector names. Second and third column: rescue from HEK293 and NIH3T3 at 30 days post transfection (d.p.t.). Fourth column: rescue from mouse genomic liver DNA of mice sacrificed at 32 days post injection (d.p.i.). The first number always indicates the number of rescue experiments were plasmid DNA could be rescued without any rearrangement from the isolated chromosomal DNA. The second number indicates the total number of performed rescue experiments. '-' indicates that no clones could be obtained by a particular rescue experiment; n.a.: not assayed.

To further analyze colony-forming efficiencies of the eight novel vectors, transfected HEK293 and NIH3T3 cells were serially diluted and selected with blasticidin. In case of HEK293 cells the results of the colony-forming assays performed are presented as *fold increase in relation to pEPI-1- [CMV-IEP]- [EGFP-IRES-BSD]-ΔMARS *(Figure 4C). Since this vector did not result in any stably selected NIH3T3 cells, the results of the colony-forming assays in NIH3T3 cells are presented as *fold increase in relation to pEPI-1- [hCMV/EF1P]- [EGFP-IRES-BSD]-ΔMARS*, (Figure 4D). In HEK293 cells, *pEPito- [CMV-IEP]- [EGFP-IRES-BSD] *exhibited a ~70 fold increased colony-forming efficiency over its pEPI-ΔMARS-control, followed by a ~45 fold increase within *pEPito- [hCMV/EF1P]- [EGFP-IRES-BSD]*, indicating a positive effect of the MARS sequence in the pEPito vector backbone on colony-forming efficiency. Vectors based on the pEPito-ΔMARS or pEPI-1 vector backbones exhibited a ~10 fold increased colony-forming efficiency as compared to the vectors based on the pEPI-1-ΔMARS vector backbones in HEK293 cells, indicating a positive effect of the CpG depleted pEPito-ΔMARS vector backbone or the presence of a MARS element on colony-forming efficiency (Figure 4C).

In NIH3T3 cells, only vectors based on the hCMV/EF1 promoter element resulted in significant numbers of formed colonies (Figure 4D): *pEPito- [hCMV/EF1P]- [EGFP-IRES-BSD] *exhibited a ~100 fold increased colony-forming efficiency as compared to *pEPI-1- [hCMV/EF1P]- [EGFP-IRES-BSD]-ΔMARS *indicating a positive effect of the pEPito vector backbone. Vectors based on the pEPito-ΔMARS vector backbones exhibited a ~10 fold increased colony-forming efficiency as compared to the vectors based on the pEPI-1-ΔMARS vector backbones indicating a positive effect of the CpG depleted pEPito-ΔMARS vector backbone on colony establishment.

### Increased expression and stability of pEPito vectors in murine liver *in vivo*

**Figure 5:**
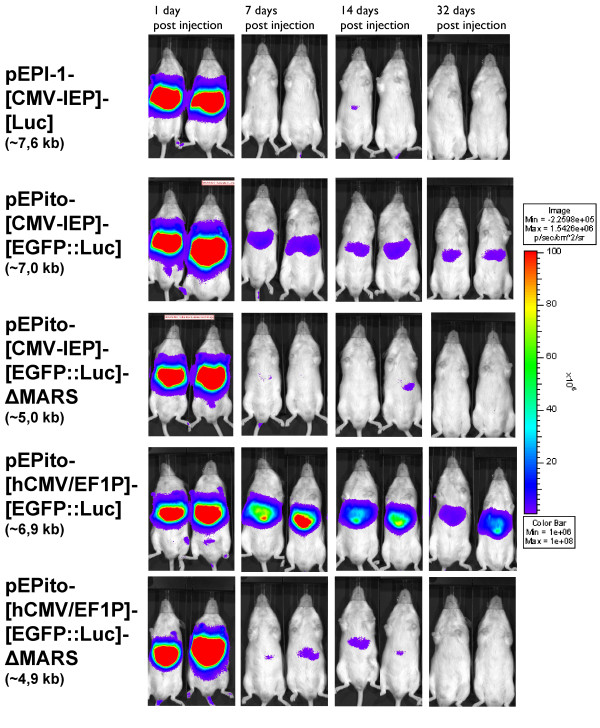
**Increased and prolonged expression of pEPito *in vivo***. Luciferase expression profiles of exemplary MF-1 mice hydrodynamically injected with the vectors #9-13 (Figure 2 as assayed by *in vivo *bioluminescence imaging after 1 (first column), 7 (second column), 14 (third column), and 32 (fourth column) days post injection. The vector pEPI-1- [CMV-IEP]- [Luc] has been previously published as pEPI-Luc [32].

The *in vivo *performance of the novel pEPito vector backbones was studied in MF-1 mice by hydrodynamic injection of 30 μg of respective vectors intravenously into MF-1 mice and the transgene expression in the liver measured over time by bioluminescence imaging as described earlier [32]. For this purpose, a set of four novel pEPito vectors were constructed containing the EGFP::Luc fusion protein as transgene transcription unit. (Figure 2E, #10-13). The vector pEPI-1- [CMV-IEP]- [Luc] (#9) has been included as additional control (recently published as *pEPI-Luc *[32]), which contains a *Firefly luciferase *(Luc) transgene instead of the EGFP::Luc fusion protein (Figure 2E, #9). The sequences of both luciferases, however, are identical. In concordance with all previous (*in vitro*) experiments, equal amounts of vector DNA have been injected, in order to avoid undesired effects of any added stuffer DNA. Luciferase activity was quantified after 1, 7, 14, 21 (data not shown) and 32 *days post injection *(d.p.i.) (Figure 5, for quantifications see additional file 3). All animals exhibited an equally strong luciferase signal on day one after injection, at later time points the luciferase signal steadily decreased. With the pEPI-1 based control vector pEPI-1- [CMV-IEP]- [Luc], and in the two MARS depleted pEPito control vectors pEPito- [CMV-IEP]- [EGFP::Luc]-ΔMARS and pEPito- [hCMV/EF1P]- [EGFP::Luc]-ΔMARS the luciferase signal is weakly detectable until 14 d.p.i., but is below detection limit at 32 d.p.i This result indicated a positive effect of the pEPito vector backbone and the MARS element on prolonged luciferase expression. In contrast, the two pEPito based vectors pEPito- [CMV-IEP]- [EGFP::Luc] and pEPito- [hCMV/EF1P]- [EGFP::Luc] exhibit a longer lasting luciferase expression until 32 d.p.i Obviously, the pEPito vector with the hCMV/EF1P promoter element exhibited the strongest *in vivo *luciferase expression 32 d.p.i., about 5 fold higher compared to the CMV-IEP in pEPito and more than a 2 log difference to pEPI-Luc indicating a positive effect of the hCMV/EF1P promoter element on prolonged transgene expression in MF-1 mice *in vivo*. After sacrificing all animals at 32 d.p.i., chromosomal DNA was extracted from their livers. In order to estimate the copy numbers of vector molecules present in liver tissues a quantitative PCR (qPCR) was performed. Vector DNA could be detected in all animals at 32 d.p.i The copy number of vector molecules as detected by qPCR varied between 0.5 - 6 vector molecules per liver cell, but did not vary significantly within the livers of the animals injected with any of the five different vector constructs, implying that no degradation (loss) of vector DNA is responsible for the decrease in the luciferase signal over time (data not shown). As indicated in Table 1, the two pEPito based vectors with MARS element, i.e. pEPito- [CMV-IEP]- [EGFP::Luc] and pEPito- [hCMV/EF1P]- [EGFP::Luc], could be rescued from chromosomal liver DNA preparations without any rearrangements at 32 d.p.i., indicating that the presence of a MARS element within the pEPito vector backbone facilitates the persistence of episomes in a "rescue"-able form *in vivo*. We did not succeed in rescuing pEPI- [CMV-IEP]-Luc.

## Discussion

In this manuscript we present a novel pEPI-vector derivative, named pEPito, with higher transgene expression levels and increased colony-forming efficiencies *in vitro*, and more persistent transgene expression profiles *in vivo*. In order to compare the novel pEPito-backbone with the backbone of the original vector, pEPI-1, a series of 13 novel vectors was constructed. Eight vectors, comprising an identical transgene transcription unit, i.e. EGFP-IRES-BSD, were generated to evaluate vector-backbone performance *in vitro*. These eight vector constructs were subsequently selected for BSD expression, present in the first transgene transcription unit that is part of the pEPI-vectors' episomal plasmid replicon (Figure 1). This is noteworthy, since all previous pEPI-1 based vectors were selected for the NPT gene, present in the second dispensable SV40-O/P driven transcription unit [1,3,4]. It is also worth mentioning, that in previous studies the expression of the first, CMV-IEP promoter driven transgene transcription unit has not been studied extensively, since main focus was laid on the analysis of the vectors' episomal replication potential, but not on transgene expression levels [3,7-9]. In these studies partial, i.e. background, integration of few pEPI-vectors was never fully excluded, but the episomal maintenance and replication was described [1-4,6-9]. When assaying all novel EGFP-IRES-BSD cassette containing vectors, the CpG-depleted, pEPito based constructs constantly performed better than the pEPI-1 based constructs, with respect to transient transfection efficiencies, transient EGFP expression levels, EGFP expression levels within stably selected cells, and colony-forming efficiency (Figure 3, 4). Since all experiments were performed by transfecting equal amounts of vector DNA - and not equal molar ratios of vector molecules,- to exclude side effects of stuffer DNA, some of the differences within the increased transient transfection efficiencies of the smaller plasmids are caused by this. pEPito-based vectors are on average some 25% smaller than their respective pEPI-1-based counterparts and as a consequence ~one third more pEPito vector molecules as compared to its direct pEPI-1 counterpart had been transfected initially. The differences in transient EGFP expression, the EGFP expression levels of stably selected mixed-clones, and the 7-70 fold increase in colony-forming efficiency are impressive and not caused by the different equimolar levels of the transfected DNA, which differ at a maximum of 33%. The known establishment efficiency for pEPI-1-replicons is about 1-5% [8]. In our modified experimental setup this efficiency was lower, considering the colony-forming assay, the initial transfection rate and cell growth, and resulted in about 0,25% of the transfected and selected cells for the pEPI-1- [CMV]-construct, and - ~6 fold higher - about 1,8% for the comparable pEPito- [CMV]-construct in HEK 293.

Increased transgene expression levels and longer lasting transgene expression *in vitro *and *in vivo *have also been reported for so-called *minicircles *which lack any residual elements necessary for bacterial propagation [33,34], or for *CpG-depleted or CpG-free vectors *that have been constructed depleted or free of any CpG motives [35,36]. It was shown, that the CpG content of a minicircles' transgene transcription unit does not affect the epigenetic silencing of its transgene [37], in contrast to epigenetic elements present in the bacterial origin of replication or backbone [38]. Chen et al. speculate that there might be a recruitment of repressive heterochromatin as inducer of a silencing complex that is initiated within bacterial vector elements [20], implying an important role of the chromatin structure for epigenetic silencing. As bacterial elements usually contain CpG motifs, the known effects of CpG-isles and the work of Chen et al. [20] are neither identical nor contradictory, but these effects might be linked at some points in our understanding. Further research has to elucidate, if and how these facts are connected. No or only weak silencing effects have been described within CpG-free plasmids [17,21,35]. Our novel pEPito vector, which consists of a rather CpG-rich transgene transcription unit within a CpG-depleted bacterial vector backbone, seems to exhibit similar expression profiles as the described minicircles and CpG-free vectors.

The CMV-IEP promoter element is frequently used within commercial plasmid vectors, since it confers robust expression of a transgene in several cell types. The expression profile of CMV-IEP, however, decreases steadily over time, which could be explained by methylation of cytosines within this promoter region [19]. For pEPI-1 based vectors methylation of cytosines within the CMV-IEP promoter region has been reported to be inhibited by the vector encoded MARS element in CHO and HaCat cells *in vitro *[39]. In contrast, Argyros et al. described silencing of a CMV-IEP promoter driven luciferase transgene within a pEPI-1 vector backbone after hydrodynamic gene delivery in mice. Within the latter study, however, the cytosine methylation of the CMV-IEP promoter element has not been investigated in detail [32]. Taken together, no cytosine methylation of CMV-IEP promoter elements within pEPI-based vectors has been observed so far *in vitro *and *in vivo*. Therefore, a possible explanation for the silencing of transgenes regulated by the CMV-IEP promoter element within a pEPI-vector backbone might be histone deacetylation as proposed by Papapetrou et al. [40].

When comparing the CMV-IEP and hCMV/EF1P promoter elements within our constructs (Figure 3, 4), it is obvious that the relative promoter strength has a strong influence on vector performance. In case of stably selected HEK293 cells, CMV-IEP promoter based vectors exhibited a stronger transgene expression - as a measure of promoter strength - as vectors based on the relatively weaker hCMV/EF1P promoter element (Figure 4A). This situation, however, is inverted in stably selected NIH3T3 mixed-clones, in which the hCMV/EF1P promoter element results in higher transgene expression levels than the CMV-IEP (Figure 4B). We assume that increased transgene expression levels reflect increased transgene mRNA transcription levels, despite this has not been experimentally verified so far.

In both, HEK293 and NIH3T3 cells, the colony-forming efficiency of vectors with a functional pEPI-plasmid replicon, i.e. all MARS containing vectors, seems to partially correlate with the relative promoter strength (Figure 4). The rather weak performance of the CMV-IEP promoter element within NIH3T3 cells has been already described by Nehlsen et al. [41]. These findings suggest, that the CMV-IEP promoter element seems to be extraordinarily susceptible for epigenetic silencing within NIH3T3 cells. For the latter cell line rapid silencing of exogenous plasmid DNA by histone deacetylation has been frequently observed [42,43]. The hypothesis presented in Chen et al. [20], i.e. the formation of heterochromatin after histone deacetylation involvement [44-47] triggered by certain bacterial elements, may serve as an explanation for the better performance of pEPito based vectors with respect to transgene expression and colony-forming efficiency within stably selected NIH3T3 cells (Figure 4B, D), since the pEPI-1 vector backbones exhibit significantly higher numbers of CpG motives (Figure 2).

The positive effect of the MARS element within the different pEPI-vector backbones can be observed best within our colony-forming assays *in vitro *and within the expression profiles of our MF-1 mice *in vivo*. In all colony-forming assays the presence of a MARS as a functional component of the pEPI-vector plasmid replicon (Figure 1) increased the number of established colonies as compared to their respective control vectors without MARS (Figure 4C, D). This could be explained by the episomal replication of respective vectors, which could be further supported by bacterial rescue experiments (Table 1). The CpG depleted backbone also influences the colony forming efficiencies. As the pEPI-replicon needs the transcription into the MARS-Element to establish an episome, the higher transgene expression of the pEPito vectors might lead to a higher chance of an establishing event, resulting in higher colony forming unit numbers. In MF-1 mice the pEPito based vectors with the MARS elements resulted in prolonged transgene expression (Figure 5). Since liver cells are quiescent cells, episomal replication of pEPI-based vectors might not serve as a suitable explanation for the prolonged transgene expression. Above all, we showed by qPCR that all five hydrodynamically delivered vectors persisted at equal copy numbers of approximately 0.5 - 6 vector molecules per liver cell. The failure of rescuing of the pEPI-1 based vector could be due to the formation of heterochromatin as discussed for the *in vitro *results before. We favour the theory, that the prolonged transgene expression profiles of pEPito-based vectors *in vivo *results from a combination of (1) reduced epigenetic silencing due to the modified bacterial vector backbone [17,20,23,35,48], and (2) the presence of the MARS which might trigger the translocation of vector molecules to sites of active chromatin (Figure 1) [8], or enhance overall transcription levels [49,50].

## Conclusions

In conclusion, we developed a CpG-depleted pEPI-vector derivative named pEPito in this study which combines the ability of episomal replication and mitotic stability described for pEPI-based vectors with the diminished tendency towards epigenetic silencing of vector encoded transgenes due to the CpG depletion of the vector backbone. In contrast to recently described minicircles, pEPito-based vectors can be propagated, modified, and amplified in *E.coli *DB3.1λpir. We could further demonstrate, that the CpG content and/or the bacterial vector backbone of any plasmid vector exhibits a strong influence on long-term transgene expression. The detailed molecular mechanism of the epigenetic silencing of vector encoded transgenes has to be further investigated. Our novel pEPito vector will have crucial implementations for biotechnological applications *in vitro*, e.g. within the fast generation of stably selected mixed-clone cells with arbitrary transgenes at low costs. Due to its ability for prolonged transgene expression *in vivo*, pEPito is also expected to serve as an improved vector for non-viral gene therapy.

## Methods

### Vector construction

The construction of the pEPI-1 and pEPI-1ΔMARS vector backbones have been described previously [1]. The individual functional elements of the vector can be exchanged by restriction digest and ligation using *Pci*I/*Nhe*I (promoter element), *Nhe*I/*Bgl*II (transgene transcription unit), or *Bgl*II/*Mlu*I (MARS). For the construction of the pEPito and pEPito-ΔMARS vector backbones, a 729 bp long DNA fragment, containing a CpG free R6Kori and a zeocin resistance cassette, of the vector pCpG-MCS (Invivogen, France) was isolated by *Pac*I restriction and gel purification. This R6Kori *Pac*I fragment was ligated to the 918 bp long CpG depleted β-lactamase gene (BLA) cassette of the vector pMOD-LucSH (Invivogen, France). The latter (BLA) cassette was PCR-amplified from pMOD-LucSH in a way that *Pac*I restriction sites were added to either ends by primer mutagenesis. For further subcloning purposes, an additional *Xho*I site was added to the 5' end of the BLA cassette. The resulting first shuttle vector (pHulk-ΔMCS) consisted of a CpG free R6Kori, a CpG depleted BLA cassette, and a *Xho*I single cutter site. A multiple cloning site consisting of the restriction enzyme sites *Xho*I, *Pci*I, *Nhe*I, *Bgl*II, *Mlu*I, and *Xho*I was generated by primer annealing and inserted into the single *Xho*I site of pHulk-ΔMCS. The resulting shuttle vector (pHulk) served as a template for the construction of all pEPito and pEPito-ΔMARS vectors.

As described above for the pEPI-1 and pEPI-1-ΔMARS vector backbones, the individual functional vector elements could be inserted into pHulk by restriction digest and ligation using *Pci*I/*Nhe*I (promoter elements), *Nhe*I/*Bgl*II (transgene transcription units), or *Bgl*II/*Mlu*I (MARS). The *Cytomegalovirus immediate early promoter *(CMV-IEP) element was isolated as *Pci*I and *Nhe*I fragment out of pEGFP-C1 (Clontech, USA). The *human CMV enhancer/human elongation factor 1 alpha promoter *(hCMV/EF1P) element was PCR-amplified out of pCPG-hCMV-Luc using 5' NheI and 3' BglII primers. For the construction of the *EGFP-BSD cassette connected via internal ribosomal entry site *(EGFP-IRES-BSD), the BSD cassette of pLenti6.2/V5-DEST (Invitrogen, USA) has been PCR-amplified by using a 5' primer that contains a *Bgl*II site and a recently described, 22 bp long synthetic IRES module [25] and a 3' primer containing a *BamH*I site. The resulting PCR-fragment containing IRES-BSD was inserted via *Bgl*II and *BamH*I into the single *Bgl*II site of pEGFP-C1 (Clontech, USA) or pEPI-1 [1]. After checking the correct orientation, the IRES-BSD transgene transcription unit could be isolated with or without MARS element from the respective vectors (Figure 1E, #2 and #1) as *Bgl*II and *Mlu*I fragments. The *EGFP-luciferase fusion protein *(EGFP::Luc) [27,28] was PCR-amplified from pEGFPLuc (Clontech, USA) using 5' NheI and 3' BglII primers. The vector pEPI-1- [CMV-IEP]- [Luc] (Figure 1E, #9) has been described earlier [32]. All 13 different vectors used within this work were constructed, propagated, and amplified in *E.coli *DB3.1λpir [30]. The integrity of vectors was verified by sequencing (AGOWA, Germany). Additional information about vector sizes and total amounts of CpG motives are provided in the last two columns of Figure 2E. Vector DNA was prepared using the QIAprep Spin Miniprep Kit (Qiagen, Germany) according to the manufacturer's instructions and stored at 4°C until usage. Freezing of the purified vector DNA was avoided to prevent shearing of the DNA.

### Mammalian cell culture and transfection

HEK293 and NIH3T3 cells were cultured in Dulbecco's modified Eagle medium supplemented with 10% FCS and 1% glutamine (Gibco, USA). For transfection experiments, 2 × 10^5 ^cells were seeded into a 6 well plate (BD Falcon, USA) 24 hours prior to the experiment. Transfections were performed using 0,5 μg vector DNA and Fugene6 (Roche, Germany) as transfection reagent according to the manufacturer's instructions.

### Flow cytometry

Two days (48 hours) post transfection the cells were trypsinized, resuspended in phosphate buffered saline (PBS) (Invitrogen, Germany), and divided into two halves. One half was used for immediate flow cytometric analysis of EGFP expression using a FACS Canto II device (Becton Dickinson, Germany). The other half was cultured in the presence of either blasticidin (7 ng/ml) or geneticin (400 ng/ml) (PAA, Austria). After 28 days of selection, stably selected (mixed-clone) cells were again analyzed for EGFP expression. EGFP signal was measured in duplicates at 488 and 567 nm to exclude autofluorescence of different cell lines.

### Colony-forming assay

For colony forming assays, transfected cells were split from 6 well plates into 75 cm^2 ^flasks at 48 hours post transfection. Splitting of cells was performed at serial dilutions (1:1, 1:10, and 1:100). After a total of 30 days of selection, with either blasticidin or geneticin, formed colonies were fixed with 4% paraformaldehyde (Sigma, Germany) in PBS, counterstained with methylene blue (Sigma, Germany) and counted.

### Isolation of genomic DNA from cell lines and mouse liver

For isolation of genomic DNA from transfected and stably selected cell lines, cells were trypsinized, resuspended in PBS, and counted. Genomic DNA was isolated from 10^7 ^cells using the QiaAMP DNA Mini Kit (Qiagen, Germany) according to the manufacturer's instructions. For isolation of genomic DNA from shock frozen mouse liver, small pieces of about 25 mg were cut on dry ice. Genomic DNA was isolated immediately using the QiaAMP DNA Mini Kit (Qiagen, Germany) according to the manufacturer's instructions.

### Bacterial rescue experiments

Bacterial rescue experiments to verify the episomal status of pEPI-based vectors within transfected and stably selected mammalian cell lines or liver tissues were performed by chemical transformation of 10 μl of isolated genomic DNA (approximately 500 ng) in chemical competent *E.coli *GT115 (Invivogen, France). Transformed bacteria were selected on LB-plates containing either ampicillin, or kanamycin depending on respective bacterial selection markers (Table 1). Plasmid DNA was prepared out of transformed bacteria using the Qiaprep Spin Miniprep Kit (Qiagen, Germany) according to the manufacturer's instructions. The integrity of the rescued plasmids was checked by restriction analysis and gel electrophoresis. For rescue experiments of cell culture materials, chromosomal DNA of stably selected mixed clones was isolated three times independently and transformed into bacteria. Resulting bacterial clones were analysed for the integrity of their isolated plasmids. In case no colonies could be obtained from the initial transformations, this procedure was repeated for two more times. For rescue experiments of liver tissue materials, the DNA of three different livers was isolated three times independently and transformed into bacteria as described above.

### Hydrodynamic plasmid injection of animals and *in vivo *bioluminescence imaging

MF-1 mice (1-2 months old, 20-25 g, male) (B&K Universal Ltd., UK) were rapidly injected, over 5-8 seconds, via the tail vein with 2.5 ml PBS containing 30 μg of each plasmid vector DNA using a 27-gauge needle. Animals were given appropriate care in compliance with institutional and UK guidelines. All animal procedures were conducted in accordance with the Animal (Scientific Procedures) Act 1986, and after appropriate local ethical review. At 1, 7, 14, 21, and 32 days after hydrodynamic injection, mice were dosed intraperitoneally (i.p.) with 300 μl of D-luciferin (Gold Biotechnology, USA) (15 mg/ml in PBS), anesthetized by isoflurane, and then imaged for bioluminescence by the IVIS Imaging 50 Series (Xenogen, USA). Bioluminescence imaging (BLI) was performed in a light-tight chamber on a temperature-controlled, adjustable stage while isoflurane was administered by means of a gas manifold at a flow rate of 2%. Images were acquired at a medium binning level (8) and a 20-cm Weld of view. Acquisition times were either 5 or 10 sec, depending on the intensity of the luminescence. The Xenogen system reported bioluminescence as photons/sec/cm^2^/sr (seradian) in a 2.86-cm-diameter region of interest covering the liver. The auto function was used to define the minimum for the scale at each time point. This value was 5% of the maximum in each case. Data were analysed by using LIVINGIMAGE 2.50 software (Xenogen, USA). Background levels of bioluminescence were 1 × 10^6 ^photons/sec/cm^2^/sr. Comparison of luciferase expression resulting from the various constructs was analysed by one-way ANOVA to assess statistical significance. A post-ANOVA multiple comparison procedure (Tukey's HSD) was further performed to assess pairwise differences on expression confirmed by ANOVA with a significance level p = 0.05.

### Quantitative PCR

The quantitative PCR (qPCR) for the detection of vector molecules within genomic DNA isolated from mouse liver was performed on an AB Prism 7500 SDS device using FastStart Universal Probe Master (Roche, USA). Vector molecules were detected using probe (5'Fam-CGCCCAACACCGGCA TAAAGA-3'Tamra) and primers (TTGGCAGAAGCTATGAAACG, GCAAC TGCAACTCCGATAAA) against the luciferase gene (Ella Biotech, Germany). For standardization, probe (5'Joe-CAAACACGAACCATCCGCCG-3'Tamra) and primers (CAGCATCAATGGCAACTTCT, GAAGATTGATCCGTGGCTTT) against the *neurogenic differentiation 1 *(ND1) gene were used. A standard curve with a luciferase containing plasmid was generated, and vector copy numbers per liver cell were calculated from the differences in cycle numbers of the internal standard (ND1) compared to the target (luciferase).

## List of abbreviations

BLA: β-lactamase; bp: basepair; BSD: blasticidine S deaminase; CMV: Cytomegalovirus; CpG: Cytosine-phosphatidyl-Guanosine; d.p.i.: days post injection; d.p.t.: days post transfection; EBV: Epstein-Barr virus; EF1P: elongation factor 1 promoter; EGFP: enhanced green fluorescent protein; EGFP::Luc: EGFP-luciferase fusion protein; G418: geneticin; hCMV: human cytomegalovirus; IEP: immediate early promoter; IRES: internal ribosomal entry site; kb: kilobasepair; Luc: luciferase; MARS: matrix attachment region sequence; MCS: multiple cloning site; NPT: neomycin phosphotransferase; ORC: origin recognition complex; Ori: origin of replication; PBS: phosphate buffered saline; qPCR: quantitative PCR; SAF-A: scaffold attachment factor A; shRNA: small hairpin RNA; SD: standard deviation; SV40-O/P: Simian virus 40 Ori/promoter; TLR: Toll-like receptor

## Authors' contributions

RH constructed all vectors described in this manuscript and performed the majority of *in vitro *experiments. OA performed all animal (*in vivo*) experiments. SPW contributed in the *in vivo *bioluminescence imaging. RPH provided supervision of all animal (*in vivo*) experiments. HJL and MO contributed in vector design and construction. TM contributed in real time PCR design and performance. MVP contributed in genomic DNA isolation of cell lines and mouse liver. JH and AB provided supervision. RH and AB drafted the manuscript. All authors read and approved the final manuscript.

## Supplementary Material

Additional file 1 **Representative FACS-diagrams corresponding to **figure 3A. This additional file depicts representative flow cytometry profiles corresponding to all bars shown in figure 3A (transiently transfected HEK293 cells). Click here for file

Additional file 2 **Representative FACS-diagrams corresponding to **figure 4A.
Description: This additional file depicts representative flow cytometry profiles corresponding to all bars shown in figure 4A (stably selected mixed-clone HEK293 cells). Click here for file

Additional file 3 **Quantification of expression profiles *in vivo***. This additional file depicts graphically the extended longitudinal expression study of the mice for up to 32 days. Luciferase expression is quantified using Xenogen Living Image software and represented as photons/sec/cm^2^/sr. Background level of light emission on non-treated animals is 1 × 10^6 ^photons/sec/cm^2^/sr. Standard error of the mean for each time point is indicated. Click here for file
