# Supplementary material for: pEPito: a significantly improved non-viral episomal expression vector for mammalian cells
Source: BMC Biotechnol. 2010 Mar 15;10:20. doi: 10.1186/1472-6750-10-20 (PMC2847955; doi:10.1186/1472-6750-10-20)
Supplement: Additional file 1 — Representative FACS-diagrams corresponding to figure 3A. This additional file depicts representative flow cytometry profiles corresponding to all bars shown in figure 3A (transiently transfected HEK293 cells). [file 1472-6750-10-20-S1.PDF]

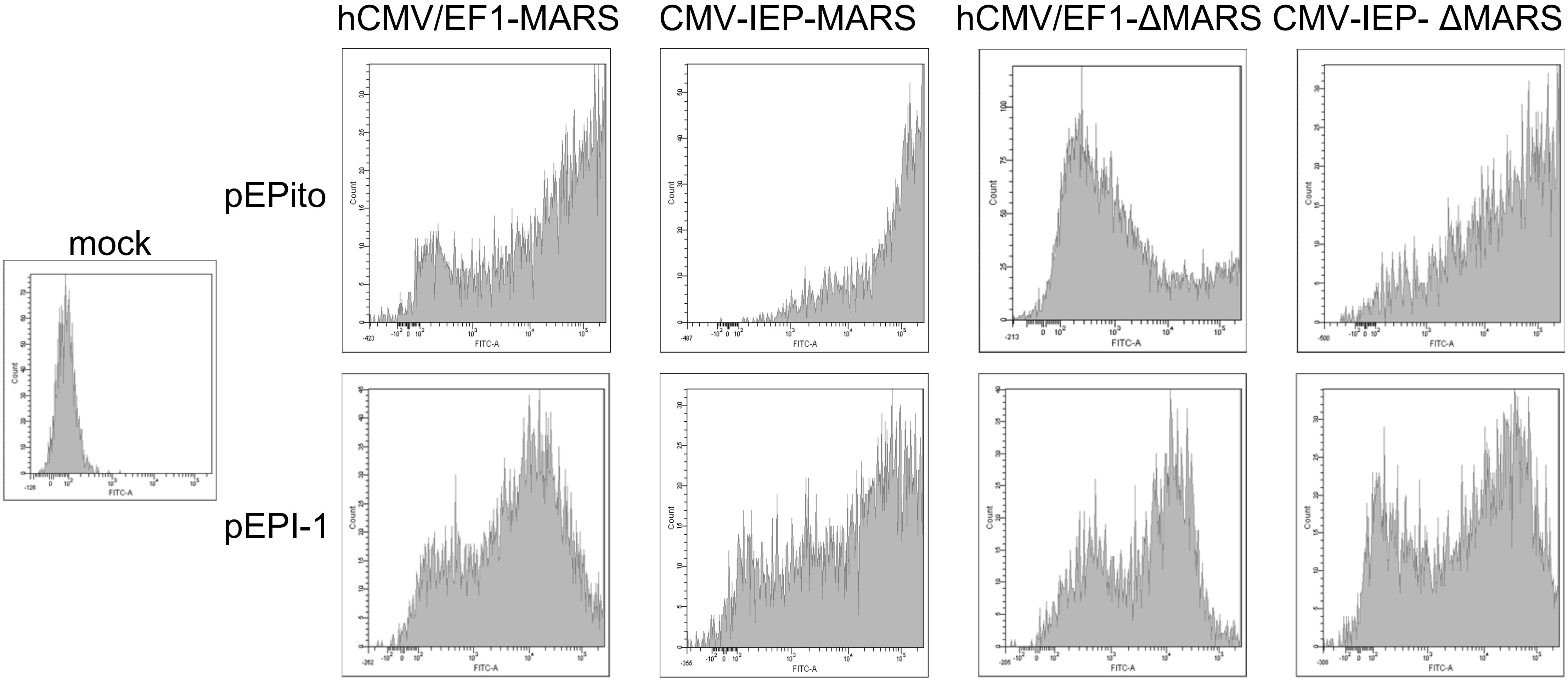

Additional file 1: Representative FACS-diagrams corresponding to figure 3A.

This additional file depicts representative flow cytometry profiles corresponding to all bars shown in figure 3A. Upper panels correspond to pEPito vectors (bars 1-4), lower panels correspond to pEPI-1 vectors (bars 5-8). At least 10.000 cells were counted. Similar results were obtained within four individual experiments.
