# Supplementary material for: pEPito: a significantly improved non-viral episomal expression vector for mammalian cells
Source: BMC Biotechnol. 2010 Mar 15;10:20. doi: 10.1186/1472-6750-10-20 (PMC2847955; doi:10.1186/1472-6750-10-20)
Supplement: Additional file 3 — Quantification of expression profiles in vivo. This additional file depicts graphically the extended longitudinal expression study of the mice for up to 32 days. Luciferase expression is quantified using Xenogen Living Image software and represented as photons/sec/cm2/sr. Background level of light emission on non-treated animals is 1 × 10^6^ photons/sec/cm2/sr. Standard error of the mean for each time point is indicated. [file 1472-6750-10-20-S3.PDF]

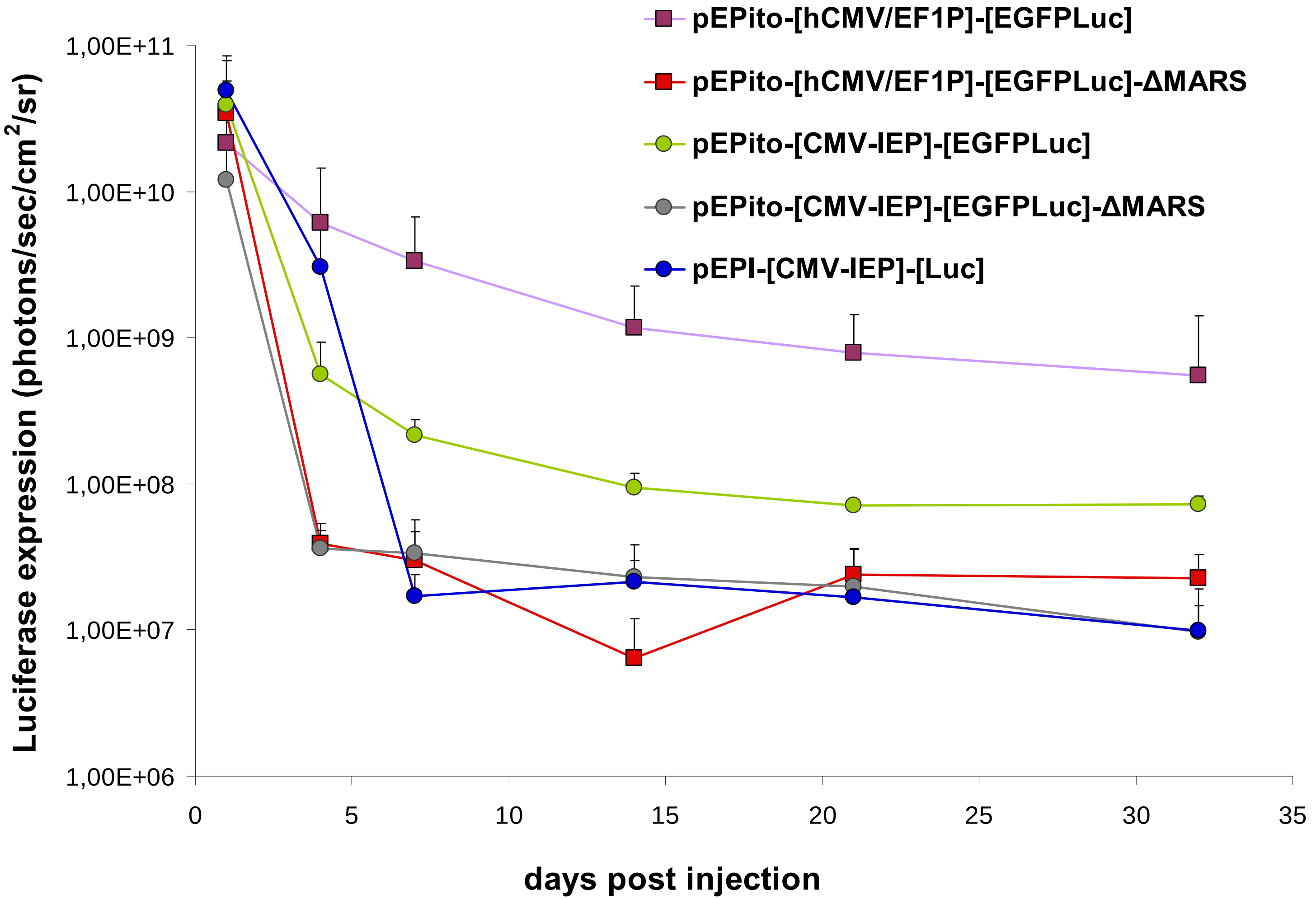

Additional file 3: Quantification of expression profiles *in vivo*.

This additional file depicts graphically the extended longitudinal expression study of the mice for up to 32 days. Luciferase expression is quantified using Xenogen Living Image software and represented as photons/sec/cm^2/sr. Background level of light emission on non-treated animals is $1x10^6$ photons/sec/cm^2/sr. Standard error of the mean for each time point is indicated.
